# Neoantigens and genome instability: impact on immunogenomic phenotypes and immunotherapy response

**DOI:** 10.1186/s13073-019-0684-0

**Published:** 2019-11-20

**Authors:** Elaine R. Mardis

**Affiliations:** Institute for Genomic Medicine at Nationwide Children’s Hospital, The Ohio State University College of Medicine, Children’s Drive, Colombus, OH 43205 USA

## Abstract

The resurgence of immune therapies in cancer medicine has elicited a corresponding interest in understanding the basis of patient response or resistance to these treatments. One aspect of patient response clearly lies in the genomic alterations that are associated with cancer onset and progression, including those that contribute to genomic instability and the resulting creation of novel peptide sequences that may present as neoantigens. The immune reaction to these unique ‘non-self’ peptides is frequently suppressed by the tumor itself, but the use of checkpoint blockade therapies, personalized vaccines, or a combination of these treatments may elicit a tumor-specific immune response that results in cell death. Massively parallel sequencing, coupled with different computational analyses, provides unbiased identification of the germline and somatic alterations that drive cancer development, and of those alterations that lead to neoantigens. These range from simple point mutations that change single amino acids to complex alterations, such as frameshift insertion or deletion mutations, splice-site alterations that lead to exon skipping, structural alterations that lead to the formation of fusion proteins, and other forms of collateral damage caused by genome instability that result in new protein sequences unique to the cancer. The various genome instability phenotypes can be identified as alterations that impact DNA replication or mismatch repair pathways or by their genomic signatures. This review provides an overview of current knowledge regarding the fundamentals of genome replication and of both germline and somatic alterations that disrupt normal replication, leading to various forms of genomic instability in cancers, to the resulting generation of neoantigens and, ultimately, to immune-responsive and resistant phenotypes.

## Background

**Fig. 1:**
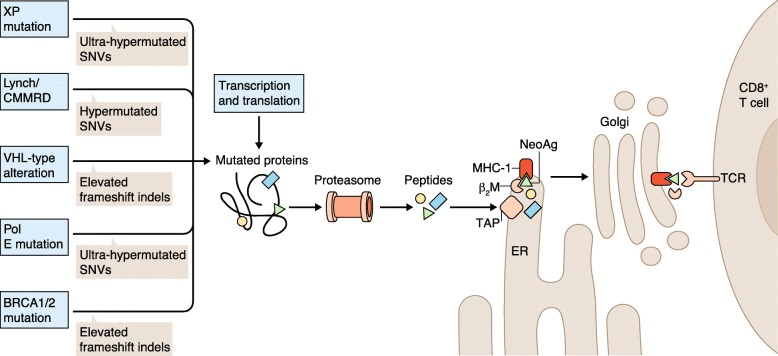
Mechanism of neoantigen presentation to T cells by MHC class 1. Genetic determinants of genome instability provide different types of alterations that sometimes change protein sequences. When these tumor-unique proteins undergo proteolysis in the proteasome, the resulting peptides are imported into the endoplasmic reticulum (ER) by the TAP (Transporter associated with antigen processing) protein. In this example, one neoantigen peptide (NeoAg; *green triangle*) is tightly bound by a complex comprising the MHC-1 protein and beta-2-microglobulin (β_2_M), and is exported to the cell surface through the Golgi apparatus. The MHC-bound neoantigen is presented on the cell surface, where it can interact with and stimulate a CD8+ T cell that expresses a corresponding T-cell receptor (TCR)

The fidelity with which our genome is copied prior to cell division is remarkable in its consistency over time. This consistency results from a variety of enzymatic DNA replication, proofreading, and damage repair functions that work in concert to minimize alterations from one cell division to the next. Nevertheless, these high-fidelity processes can become compromised by a variety of genomic alterations that subsequently result in the development of cancer, in which the normal genome-wide mutation rate becomes accelerated. Often, this consequence is due to inherited or de novo alterations in the germline that impact the proper function of enzymes that are involved in these processes, leading to different manifestations of genome instability. Because the enzymatic functions that normally ensure genome replication fidelity are altered, the resulting errors can lead to secondary, somatic alterations of several types that may change protein-coding sequences in the genome. When alterations occur in cancer-related genes, a progression to malignancy results. Alternatively, mutations may occur in so-called ‘passenger genes’ that have no link to cancer onset or progression. In either case, the alterations that have resulted (directly or indirectly) from genomic instability in genes that are transcribed and translated, encode novel peptide sequences that are unique to the cancer cell. During normal protein degradation, these novel peptides may be bound by major histocompatibility complex (MHC) proteins that present them on the cell surface as ‘neoantigens’ (i.e., tumor-specific peptides that can be recognized by the immune system as non-self, making the cancer cells targets for destruction). This process is summarized in Fig. 1.

Many years of basic cancer immunology research have delineated the mechanisms by which cancer cells suppress this anti-cancer immune response through tolerance and immune suppression mechanisms. However, immune-based cancer therapies (‘immunotherapies’) such as checkpoint blockade inhibitors, which were inspired in their formulation by the research that revealed immune checkpoint suppression, have now established that the therapeutic (antibody-based) inhibition of immune suppression checkpoint proteins allows the immune system to become engaged and to eradicate the cancer cells. Hence, this review will examine knowledge accrued to date that links genome instability (in its many forms) to the generation of neoantigens and to treatment response or resistance to different immunotherapies. Taken together, this review explores how genomic instability and its consequences are emerging as a key clinical consideration in cancer precision medicine.

### Fundamentals of genome replication

The human genome is large and repetitive, yet each human cell division is accompanied by the highly accurate replication of approximately six billion base-pairs of DNA. Fidelity in replication is a critical component of this process, and both polymerase proofreading by polymerase epsilon and delta and the mismatch repair (MMR) system improve basic replication fidelity by about 100-fold [1–3]. In normal chromosomal replication processes, replication errors inevitably escape correction and provide a baseline rate of somatic mutations, which accumulate in the resulting cell lineage over time and with increasing age. When these fundamental aspects of replication fidelity are disrupted by functional alterations in MMR system enzymes or in the proofreading polymerases, as well as in the settings of other base excision and homologous repair defects, the baseline mutation rate at genome replication in the context of cell division is elevated to differing degrees [4], and genomic instability results. Such cancer susceptibility defects can be acquired by individuals through either inheritance or spontaneous mutation. Historically, the diagnosis of conditions that involve genomic instability, such as Lynch syndrome or the presence of BRCA1/2 or xeroderma pigmentosum (XP) defects, has involved the use of imaging-based cancer screening at an earlier age and with increased frequency than that appropriate for the general population. Other assays, such as colonoscopy, are also used to detect early-onset cancers. In the present day, however, the different types of genomic instability imparted by replication defects—including mismatch repair, base excision, and homologous end joining—increasingly have implications for cancer treatment and for treatment response, as this review explores. Further resolution to the nuanced impact of genomic instability is also emerging, as we realize that different genomic alterations elicit different responses to immune checkpoint blockade therapies.

### Germline and somatic contributors to genomic instability

Basic research to characterize the enzymatic machinery of DNA replication [5] and to define genetic syndromes that result from fidelity defects in DNA replication [2] has helped to elucidate the fundamental processes involved in eukaryotic chromosomal replication, to identify the enzymes responsible for replication fidelity and their variants, and to develop assays that diagnose these syndromes [6]. The specific details of DNA replication fidelity mechanisms and their associated defects are beyond the scope of this review, but numerous genes and their pathogenic alterations have been catalogued in terms of their contribution to genomic instability. In general, these genes and mutations can be altered in the germline (by either inherited or de novo mechanisms), in the somatic tissue genome, or in both. Inherited mismatch repair defects can be seen in Lynch syndrome, which is characterized by autosomal dominant inheritance of heterozygous pathogenic germline mutations in one of the MMR genes (*MLH1*, *MSH2*, *MSH6*, or *PMS2*) [7]; in Lynch-like syndrome, which results from double somatic mutations in one of the MMR genes; and in constitutional MMR deficiency syndrome (CMMRD), an autosomal recessive disorder caused by biallelic pathogenic germline mutations in MMR genes [8].

Lynch syndrome is the most prevalent of these mismatch repair defects at an estimated 1 in 279 individuals [9], although Lynch-like syndrome has recently been estimated to have a similar incidence [10]. The proportions of mutations in each of the Lynch syndrome genes are *MLH1* (40%), *MSH2* (34%), *MSH6* (18%), and *PMS2* (2%), with the cancer risk varying depending on the gene involved. Sporadic MMR deficiency also occurs, typically as the result of hypermethylation of the *MLH1* promoter, which causes loss of MLH1 protein expression [11]. This sporadic form of MMR deficiency is a common driver of colorectal and endometrial cancers, identified in 69 and 94% of *MLH1* and *PMS2* non-mutated cases, respectively. Germline pathogenic mutations in *POLD1* and *POLE* are found in the exonuclease domain and have been documented in familial cancer syndromes [12–19], although they occur at quite low population frequencies (≤ 0.002). BRCA1, BRCA2, and PALB2 proteins are components of the protein complex that effects DNA repair at double-stranded breaks (DSBs), and alterations to the genes that encode these proteins have been linked to inherited breast, ovarian, and endometrial cancer susceptibility [20–25]. The incidence of *BRCA1/2* inherited mutations has been estimated at 1 in 400 but this is subject to ancestry considerations, as has been well documented in certain populations. Similarly, these genes also can be mutated in the somatic genome and contribute to an overall increase in insertion and deletion mutations genome-wide.

In summary, genetic contributions to genome instability are inherited, sporadic, and somatic in nature, often combining to drive the development of cancer with a variety of impacts upon genome instability that are detectable by a variety of diagnostic approaches. Identifying these alterations has meaning in the contexts of cancer predisposition, monitoring, and early detection, as well as for indicating newer types of cancer therapy that can engage each patient’s immune system in eradicating the disease.

## Approaches for the detection and diagnosis of genomic instability

Historically, MMR defects have been diagnosed using a combination of PCR and the sequencing of specific microsatellite regions to detect microsatellite instability (MSI) and immunohistochemistry to assess the altered expression levels of MMR proteins within the tumor that might correlate with a diagnosis of high MSI. These assays were based on the understanding that defective mismatch repair leads to uncorrected DNA polymerase errors at mono- and dinucleotide microsatellite loci during genome replication, and could be correlated to methylation-based epigenetic silencing of MMR genes that leads to reduced levels of the encoded proteins. Although these assays were once considered adequate for the diagnosis of mismatch repair defects, recent large-scale studies, aimed at characterizing somatic and germline alterations in tumor vs normal comparisons by the use of massively parallel or next generation sequencing (NGS), have led to an enriched understanding of the numbers and types of alterations that occur in genes associated with genome instability. In particular, NGS-based assays to detect and diagnose genomic instability are achieving increased resolution relative to previous approaches. Hence, our understanding of the consequences of genomic instability, with regard to how they may engage the immune system and determine responses to new immune therapy modalities, is described herein.

These NGS-based studies of tumor and germline DNA have illustrated that genes encoding replication, proofreading, or DSB repair proteins are concurrently altered on both alleles by loss of heterozygosity, monoallelic deletion, epigenetic silencing, or mutation in tumor tissue, thereby acting in concert with the inherited defect [26]. Moreover, certain types of genomic instability impart a specific sequence-based mutational signature that can be detected by appropriate computational analysis of NGS data [27, 28]. For example, Nik-Zainal’s group has used the specific mutational signatures from NGS whole genome sequencing of breast cancers to detect homologous repair defects in BRCA-complex-mediated genomic instability that predict patients who are likely to respond to poly ADP ribose polymerase (PARP) inhibitor therapy [29].

Beyond detecting specific genomic alterations, the accompanying mutational load or tumor mutational burden (TMB) can be quantified using somatic analysis pipelines [26, 30]. The important roles played by proteins that are involved in maintaining proper DNA replication fidelity or DSB repair dictate that cancers with defective replication/proofreading or DSB repair have an elevated mutation rate when compared to cancers from the same tissue site without alterations to these proteins [27]. As these alterations, together with the normal stochastic background mutations that occur over time, impact protein-coding genes in the cancer genome and may change amino acid sequences, the resulting changes are referred to as the ‘mutanome’. In particular, somatic alterations that change amino acid sequences create unique proteins that may, upon intracellular degradation, be bound by MHC molecules that present them on the cell surface, as discussed earlier. The interaction between CD8+ T cells and MHC-presented neoantigens elicits T-cell-specific recognition of each ‘non-self’ neoantigen peptide, thereby allowing the patient’s immune system to distinguish cancerous from normal cells. Neoantigens result from somatic changes, including simple point mutations that substitute a different amino acid, insertions or deletions of nucleotides that shift the open reading frame, and inversions, translocations, or other structural alterations that result in protein fusions.

Therefore, the NGS-based evaluation of cancers using analytical approaches that are capable of detecting these types of alterations both extends and refines the information available from the conventional immunohistochemistry (IHC), PCR and sequencing, or microarray-based chromosomal instability (CIN) assays that are used in the clinical diagnosis of genomic instability (Table 1). In effect, a broad-based NGS assay (exome or whole genome) provides greater resolution of the underlying germline and somatic defects and identifies the genomic consequences (the mutanome) that results from these defects, obviating the need for multiple assays to elucidate the underlying cause of each type of defect (methylation changes, mismatch repair defect (MMRd), or DSB repair). Beyond the diagnostic assay of genome instability, the identification of the tumor-unique neoantigens that are created by various defects in replication fidelity is emerging as an important therapeutic indication, both for predicting likely response to checkpoint blockade therapy and for designing personalized vaccines.

**Table 1:** Comparison of different assays used to detect mismatch repair defects and other predictors of immune therapy response or resistance

Technique or assay	Description	Attributes	Deficiencies
MSI-PCR	PCR-based amplification of known microsatellite loci, gel electrophoresis, and software scoring to detect instability as a multiple-band pattern in amplicons	Focused test with rapid turn-around time	Interpretation difficulties, limited to MSI diagnosis, no information on genetic source of MMRd
dMMR/IHC	Antibody-based staining of FFPE sections from tumor, followed by microscopic examination and scoring to detect MMR proteins (MSH2, MSH6, MLH1, PMS2)	Focused test using a conventional pathology approach, inexpensive, rapid turn-around time	Evaluates the end result of MMR protein depleting alterations only, subject to inter-individual interpretation
CIN/FISH	Hybridization of centromere-specific fluorescent probes to chromosomal spreads, microscopic scoring of centromeric counts to detect aneuploidy	Genome-wide evaluation of chromosomal instability	Counting-based evaluation that is subject to inter-individual interpretation variability
MMR/MSI-NGS panel	NGS of genes for MMR proteins, mutation detection and annotation of pathogenic variants	Focused evaluation of mutations across known MMRd genes	Insensitive to large-scale alterations such as CNVs, insufficient breadth for TMB or neoantigen prediction
NGS/WES	NGS of all known coding exons of genes, mutation detection and annotation of pathogenic variants in genes	Unbiased evaluation of mutations across all coding genes, MSI evaluation (added probes), neoantigen prediction, TMB enabled	Mutational signature calculation may be compromised by lack of breadth
NGS/WGS	NGS of whole genome libraries from cancer and matched normal DNA, comprehensive variant detection, and annotation of variants	Unbiased evaluation of mutations across all coding genes, MSI evaluation, neoantigen prediction, TMB, mutational signature enabled	Expensive to generate sufficient coverage from low cellularity tumors

*CIN* chromosomal instability, *CNV* copy number variant, *dMMR* deficient mismatch repair, *FFPE* formalin-fixed paraffin-embedded, *FISH* fluorescence in situ hybridization, *IHC* immunohistochemistry, *MMR* mismatch repair, *MMRd* mismatch repair defect, *MSI* microsatellite instability, *NGS* next generation sequencing, *TMB* tumor mutational burden, *WES* whole exome sequencing, *WGS* whole genome sequencing

## Genomic instability and neoantigen generation

### Neoantigen prediction

The use of NGS data and computational analyses to identify genomic alterations in the DNA of cancers has been reviewed elsewhere [31]. These approaches now constitute the first step in identifying which alterations change the amino acid sequences of the encoded proteins, possibly resulting (following intracellular proteolytic degradation) in the production of novel peptides that have a strong predicted differential binding affinity for MHC molecules. The transition from detecting alterations to predicting neoantigens is typically accomplished by a series of computational steps that produce in silico tiled peptide sequences around the altered amino acid sequence of each novel peptide predicted in the mutanome. Similarly, NGS data are evaluated to type the MHC proteins that are encoded by each patient’s germline, using specialized data analysis approaches that are necessitated by the hypervariable nature of these loci [32, 33]. The process by which each novel peptide is evaluated for MHC-binding strength uses one of several published methods, each of which calculates the binding affinity of each novel peptide in the context of the human leukocyte antigen (HLA) proteins for that patient (using a neural net or other machine learning-based predictor) and compares it to the binding affinity of the native peptide. Following these calculations, filtering of the list of putative neoantigens uses RNA expression data from the tumor to identify which of the proposed strong binding peptides are expressed by the tumor. Filtering exome data eliminates potential false-positive results that are caused by a lack of NGS data coverage of the normal sample, or other contributors to false positivity [34]. A multitude of nuances are associated with the identification of potential neoantigens from NGS data and several pipelines have been developed to facilitate these analyses [34–38].

Hence, neoantigen prediction from NGS data produces two potentially valuable types of information: (i) the numbers and classes of neoantigens (e.g., MHC class 1 and/or class 2 binders); and (ii) the peptide sequences that could potentially be used in personalized vaccines aimed at eliciting neoantigen-specific T-cell responses [39]. Predictably, frameshift insertions or deletions [40, 41], exon skipping events, and protein fusions [42–44], although certainly more rare than point mutations, produce significantly altered peptides, which often have higher predicted affinity for MHC molecules than peptides that contain amino acid-substitution mutations. Neoantigens that are derived from ‘noncoding’ sequences in the genome have also been reported; these are predominantly non-mutated, aberrantly expressed transcripts such as endogenous retroelements [45]. One commonly cited critique of computational approaches to neoantigen prediction is the high false-positive rate and the concern that these in silico predictions are missing important neoantigens. Several groups are attempting to address these challenges by adding mass-spectrometry-based evaluation of isolated MHC proteins from cancer samples that provide an inventory of peptides bound to MHC. These data are then compared to the corresponding computationally predicted neoantigens to differentiate true-positive from false-positive predictions. Over time and with increasing data of this type, such a dataset could be used to train a machine-learning-based algorithm to further refine in silico predictions prior to the use of neoantigens in a personalized vaccine approach [46–48].

### Immunotherapeutics and neoantigens

The connection between genomic instability and neoantigen generation is pertinent to therapeutic cancer treatments known as immune checkpoint blockade inhibitor therapies. These antibody-based therapies were the products of basic cancer immunology studies carried out in the 1990s and early 2000s that identified mechanisms, based on immune checkpoints, by which tumors evade targeting and elimination by the host immune system [49, 50]. Checkpoint proteins are typically involved in immune tolerance, preventing indiscriminate immune system attack, but several inhibitory immune checkpoint proteins that are expressed by cancer cells promote immune tolerance and permit tumor growth. Targeting these proteins with antibody-based drugs can remove the immune tolerance and permit T-cell targeting, resulting in cancer cell death [51, 52]. A general principle is that the greater the number of mutations or neoantigens present, the more likely it is that responses will be elicited from multiple, tumor-specific, T-cell populations in the context of checkpoint blockade therapy. This general principle has been somewhat borne out in clinical trials of different checkpoint blockade inhibitors, although it is certainly the case that some patients with low tumor mutational burden (and few neoantigens) also have responded to this type of treatment with tumor regression.

Several early clinical trials of immune checkpoint blockade inhibitors that were conducted in typically high mutational load tumors (such as melanoma and lung adenocarcinoma) used correlative genomic studies of tumor tissue from enrolled patients to identify a link between high TMB (> 10 mutations/Mb) and therapeutic response [53–55]. Unlike cancers with germline or somatic defects that lead to genome instability and elevated mutation rates, these cancers develop due to exposure to environmental mutagens that result in increased TMB (UV radiation from sunlight and cigarette smoke). Importantly, the observed connection of TMB to immune checkpoint response led to the hypothesis that patients with MMRd cancers, indicated by MSI ‘high’ diagnoses, might also respond to these therapies. The definitive clinical trial of checkpoint blockade therapy in MMRd cancers was initially published in 2015 [56] and indicated a trend toward therapeutic response to pembrolizumab, an anti PD-1 therapy, in MSI high/MMRd cancers. The results of the phase 2 trial, in which patients diagnosed with MMR-deficient cancers in many different tissue sites were enrolled, were reported in 2017 [57]. This phase 2 trial provided the registration data that resulted in FDA approval of pembrolizumab for all cancers with a clinical diagnosis of MSI high cancer from any tissue site. Subsequent trials and manuscripts reporting the results of immune checkpoint blockade treatments in the context of different underlying types of genomic instability are summarized in Table 2.

**Table 2:** Association of genome instability, alterations and immune therapy response

Source of genome instability	Mutational profile/burden	Neoantigen load	Response to immunotherapy	References
POLE mutation (germline or somatic)	Single nucleotide variants (SNVs)/ultra-hypermutated	High load	Checkpoint blockade responsive	[58, 59]
BRCA1/2 mutation (germline)	Frameshift indels/elevated proportion vs SNVs	Medium load/elevated number of strong binders	Checkpoint blockade responsive	[60]
Lynch syndrome (MSH1, MLH2, MLH6, PMS2)	SNVs and indels/hypermutated	High load/elevated number of strong binders	Checkpoint blockade responsive, personalized vaccine responsive	[57, 61]
VHL, SETD2, BAP1, KDM5C, FHIT defects	Frameshift indels/elevated proportion vs SNVs	Medium load/elevated number of strong binders	Checkpoint blockade responsive	[40, 62, 63]
Xeroderma pigmentosum defect (DDB2, ERCC1, ERCC2, ERCC3, ERCC4, ERCC5, POLH, XPA, or XPC)	SNVs/ultra-hypermutated	High load	Checkpoint blockade responsive	[64]
Constitutional mismatch repair deficiency syndrome (CMMRD): biallelic germline mutations in Lynch syndrome genes (MLH1, MSH2, MSH6, PMS2)	SNVs/hypermutated	High load	Checkpoint blockade responsive	[65, 66]

Similarly, patients with advanced-stage melanoma have been treated in early-phase clinical trials of neoantigen-based vaccines (NCT00683670, NCT01970358, and NCT02035956), which used the genomic approaches outlined above to identify neoantigens. The neoantigens were utilized to construct patient-specific, multi-epitope vaccines using different vaccine platforms, including dendritic cell vaccines, long peptide vaccines, and RNA-encoded neoantigen vaccines. The three studies published to date have demonstrated that neoantigen-specific T-cell populations were elicited in response to some of the vaccine-specified targets [67–69]. In two recent studies, patient responses were more durable when the vaccine was combined with an immune checkpoint blockade inhibitor [68, 69]. A fourth study also has evaluated the neoantigen vaccine approach in adult patients with glioblastoma, demonstrating that patients who did not receive dexamethasone had increased infiltration of neoantigen-specific T cells into their tumors following vaccination [70].

## Genomic instability, neoantigens and immunotherapy response

### Modeling genomic instability in preclinical mouse models informs human cancer studies

Historically, cancer and cancer therapies have been studied preclinically in mouse models by introducing alterations in cancer-associated genes into the mouse genome, and then observing the development of cancer and its response to selected therapies. However, most genetically engineered mouse cancer models have a limitation in the context of neoantigens and immunotherapeutic response studies because their cancer genomes have few mutations. Thus, the cancers that are induced in these mouse models do not share the mutational burden seen in human cancers, including those impacted by genomic instability. One exception is the methylcholanthrene (MCA)-induced mouse sarcoma model that, similar to human melanomas, has an environmental contributor to its high mutational load. In this case, the mouse cancer was generated by treatment with the chemical carcinogen, MCA. Early studies of the MCA sarcoma model illustrated a high TMB, and in silico neoantigen prediction algorithms were able to identify neoantigenic peptides with strong MHC binding (relative to that of wild-type peptides), which resulted from amino acid sequence changes that were unique to the cancer. Further studies provided evidence of tumor elimination resulting from treatment of MCA sarcomas with a neoantigen-directed vaccine and immune checkpoint blockade inhibitors [51, 71]. The results from this carcinogen-induced cancer model reflect those from the human studies cited earlier with respect to the response of UV-associated melanomas to a combination of neoantigen vaccines and checkpoint blockade treatment. A more recent study from Schreiber’s group builds upon these initial discoveries by focusing on the importance of MHCII restricted neoantigens in the vaccine-mediated immune response to cancers [72]. Here, MHCI and MHCII neoantigens from the MCA sarcoma model (mLAMA4 and mITGB1, respectively) were introduced either alone or in combination in an oncogene-driven sarcoma (KP) that lacks mutational neoantigens. Checkpoint blockade treatment in mice with contralateral tumors that expressed either both MCHI and MCHII neoantigens or only the MHC1 restricted neoantigen eliminated the former but not the latter. This result indicates that optimal anti-tumor responses to checkpoint blockade require the expression of both MCHI and MCHII neoantigens, which may have implications for human patients’ responsiveness to immunotherapies.

Recently, an elegant study by Bardelli’s group utilized transient Cas9 editing to knock out *Mlh1*, thereby inducing mismatch repair defects in mouse cancer cell lines [73]. This defect allowed the cell lines to grow into tumors in immunocompromised mice, and these tumors were subsequently transplanted into immunocompetent mice. The transplanted tumors were responsive to immune checkpoint blockade treatments, similar to human tumors exhibiting mismatch repair defects. Further genomic analysis of these *MHL1*-defective tumors as they grew over time in the immunocompetent mice demonstrated an increased and evolving neoantigen burden, indicating that DNA repair inactivation results in the continuous emergence of neoantigens in vivo. More recently, this group further investigated the longitudinal properties of neoantigen presentation by 45 colorectal cancer cell lines—including *POLE*-mutated, MSI-high, and microsatellite-stable examples propagated both in vitro (cell culture) and in vivo (xenografts)—and by patient-derived xenografts. Each example taken from the serial passage was evaluated by exome sequencing and RNAseq, with accompanying identification of single nucleotide variants (SNVs) and indels, as well as by neoantigen prediction. The results of this work illustrated that during cell-line growth in culture, in mouse xenografts or in patient-derived xenografts, MSI-high cells or cells with *POLE* mutations (with accompanying MSI-high genotypes) yielded an evolving neoantigen landscape over the longitudinal analysis. The MSI-high cells produced more frameshift indel neoantigens than did the *POLE* cells, which predominantly produce SNV neoantigens. RNA analysis of these samples illustrated that hypermutated colorectal cancer cells restrict host detection by selectively downregulating components of the neoantigen presentation process [74].

These results can be extrapolated to the human setting of mismatch repair defects, where cancers continue to occur over time with novel mutations and an accompanying high neoantigen burden [73]. A recent single-patient study in the setting of a germline *POLE* defect parallels the results of Bardelli’s group: comparisons of a primary glioblastoma to two spinal drop metastases (one prior to and one following checkpoint blockade inhibitor response) indicated an evolving neoantigen burden in each cancer sample studied [58]. Taken together, these results imply that checkpoint blockade therapies may have a protective or preventative efficacy in patients with underlying genomic instability resulting from MMRd, and encourages clinical trials to explore the use of these therapies in cancer prevention trials for patients who are highly likely to develop cancer.

### Pan-cancer evaluation of neoantigens and immunotherapy response

Large-scale genomic studies of human cancers such as The Cancer Genome Atlas (TCGA) have provided the landscape of somatic and germline alterations, along with the transcriptome and methylome profiles, that largely define human cancers. More recently, computational approaches have emerged that are able to characterize the immune cell types that infiltrate tumors on the basis of the RNA sequencing data provided by studies such as TCGA. Using data available for the 20 solid cancer types included in TCGA, Trajanoski and colleagues recently published their computational evaluation of the pan-cancer immunogenome [75]. Here, the composition and functional orientation of the immune infiltrate, both cytotoxic and immunosuppressive, and the expression of neoantigenic peptides emerging from both somatic point mutations (SNVs) and cancer germline antigens were evaluated for 20 tumor types. The results of this study have been deposited into a web-accessible relational database called TCIA (https://tcia.at/). The findings have important implications that relate to the observation, across multiple studies of different tumor types and different immune checkpoint blockade therapies, that not all patients with an elevated tumor mutation burden, regardless of its origin, respond uniformly to this type of therapeutic intervention. In particular, Trajanoski and colleagues determined that, although elevated neoantigen burden resulting from increased mutational load had an impact on tumor immunogenicity, this was only one of several tumor-intrinsic factors that combined with tumor-extrinsic factors (such as T-cell trafficking, the presence of immunomodulatory chemokines, and the infiltration of effector and immunosuppressive tumor-infiltrating lymphocytes) to determine the overall immunophenotype of a cancer [75]. These results invoke similar findings from studies of colorectal cancer immunity, including the impactful concept of ‘Immunoscore’ that emerged from Galon’s group [76] and the subtype-specific nuances of immunogenicity in colorectal cancer patients with MSI and *JAK1* mutations [77]. Immunoscore is based on the quantification of cytotoxic and memory T cells in the core of the tumor and its invasive margin, and has been shown to be a clinically useful prognostic marker. In the colorectal cancer study [77], the combination of specific gene expression subtyping (which yielded four consensus molecular subtypes) and genomic analysis (which identified the presence of loss-of-function *JAK1* mutations), not neoantigen load, best predicted which MSI-high patients had the highest immune infiltration and best prognosis. In other words, predicting response to immune checkpoint blockade therapy is highly complex and requires the quantification of different variables that may be tissue-site specific.

A second pan-cancer study [40] explored focused insertion and deletion alterations (indels) and their contribution to the immunogenic phenotype. Here, cancers of 19 of the solid tumor types sequenced by TCGA were evaluated for predicted neoantigens on the basis of their exome sequencing data, as described earlier. In certain tumor types, data were available to evaluate associations between indel burden and treatment response for different immune checkpoint inhibitor therapies. This study revealed that renal cell carcinomas had more than double the median proportion of indels compared to all other cancer types, with an enrichment of high-affinity predicted neoantigens three times that of non-synonymous point mutations. The derived neoantigens were nine times enriched for specific binding compared to non-synonymous point-mutation-encoded peptides. Correspondingly, the authors determined that responses to checkpoint blockade inhibitor therapies across three separate melanoma clinical trial cohorts [55, 78, 79] were significantly associated with frameshift indel counts, which was a better predictor of response than were non-synonymous point mutation counts in two of the three studies.

### HRDs and immunotherapy response

Several recent studies have further explored the relationship between genomic instability, immune cell infiltration, and, in some cases, response to immune checkpoint blockade in various tissue sites. One driver of such studies is the observation that not all patients with diagnosed MMRd cancers respond to these immunotherapies and, conversely, some patients with negative MMRd-assay results (based upon widely utilized diagnostic assays such as IHC and PCR-based MSI testing) do respond. The latter observation may be due to the type of assay used to diagnose MMRd cancers, as discussed below, because not all assays are equally sensitive. The former may be due to differences in the initiating genome alteration that drives mismatch repair defects, not all of which are equal in their impact, as also described below. Further, as explored above, other factors beyond the presence of mismatch repair defects determine treatment response or lack thereof.

In advanced prostate cancer, where two clinical trials testing response to immune checkpoint blockade in unselected patients have failed [80, 81], a report from de Bono and colleagues evaluated the diagnosis of MMRd using a variety of assays including IHC, MSI by PCR, MSI by targeted panel NGS of MMR pathway genes, and MSI by exome sequencing (WES) assay [82]. Their results showed that the PCR-based assay of MSI was more likely to give discordant (presumed false-positive) results when compared to the results of the NGS-based tests. This result indicates that not all assays for MSI detection and MMRd diagnosis are equal in diagnostic yield. Further, this study determined that prostate cancers with MMRd diagnosed by IHC or PCR-based MSI testing often, but not always, had corresponding higher mutational loads and MSI-positive results when tested by NGS. The associated analysis comparing immune cell infiltration via RNAseq deconvolution from 168 advanced prostate cancers in comparison to MMRd testing results demonstrated no positive association between total immune infiltrate and either overall mutation load (TMB) or MSI positivity as determined by targeted panel NGS assay, although MMRd mutational signature did correlate positively to higher inferred immune cell infiltration. Further analysis of mRNA expression for 762 immune-related genes in relation to MMRd status identified 24 genes whose expression was consistently correlated with MMRd diagnosis, and indicated that mismatch repair deficiencies associate with a more complex immune infiltrate, including the upregulation of genes associated with dendritic cells, macrophages, or myeloid cells and T cells. Taken together, this study indicates that a subset of lethal prostate cancers exhibit MMRd at diagnosis, that different assay methods can yield different diagnoses, and that only a proportion of diagnosed advanced prostate cancers have corresponding high TMB and stain with PD-L1 IHC. Hence, the sub-classification of advanced prostate cancer using NGS-based methods and evaluation of immune infiltration levels may better stratify patients who are likely to respond to immune checkpoint blockade treatments.

A separate study, which involved only immunohistochemistry-based analysis of endometrial cancers with a PD-L1 antibody, focused on comparing samples from patients with Lynch syndrome or *MLH1* promoter hypermethylation (MLH1hm) with MMR-intact patient samples [83]. The PD-L1 expression results for LS, MLH1hm, and MMR-intact tumors indicated that the tumor cells in LS endometrial cancers had the highest expression of PD-L1, followed by MLH1hm and then MMR-intact samples. Hence, the potential benefit from PD-1 or PD-L1 therapy might vary depending on the molecular mechanism driving MMRd.

Methylation-based silencing of homologous DNA recombination genes was recently reported in squamous cell histology cancers, including head and neck, cervical, and lung cancers [84]. Here, by extensively comparing the methylation in all homologous recombination genes to the IHC-based expression of CTLA-4 and PD-L1, the authors determined that squamous cell cancers hypermethylate *XRCC3* and *RAD51B* and (in correlation) have elevated expression of the two immune checkpoint genes. Interestingly, the hypermethylation status of these DSB-repair genes (*XRCC3* and *RAD51B*) led to elevated PD-L1 expression, a result that is discordant with the aforementioned result of a hypermethylated *MLH1* promoter in endometrial cancers by Sloan et al. [83]. On the basis of these two studies, it appears that the level of hypermethylation of different genes in mismatch and DSB-repair defects may be tissue-specific.

Mutation-driven genomic instability occurs in *POLE-* or *POLD1-*mutated cancers, where the levels of mutational burden (based on SNVs) in *POLE* exonuclease domain mutated cancers are extremely high. A recent study of the timing of *POLE* mutations established that these changes occur early in carcinogenesis and are detectable in preneoplastic lesions of both endometrial and colorectal cancers [85]. Correspondingly, evidence of CD8+ T-cell infiltration was also identified in the precursor lesions, lending credibility to the idea that these mutations occur early in the transition from normal to cancer cell, and that the neoantigens resulting from *POLE*-driven genome instability recruit immune cells that predicate the high amount of immune cell infiltration observed in resected tumors of both endometrium and colon or rectum. These findings have potentially important implications for the treatment of patients diagnosed with *POLE*-mutated cancers and corresponding ultra-high mutation levels that may vary depending upon tissue site.

A similar premise of evaluating immune involvement during progression from normal cells to cancerous lesions was recently reported in squamous cell carcinoma of the lung (SCC). Here, Galon’s group used gene expression data and multispectral imaging to characterize and compare biopsies representing nine stages of SCC development [86]. SCC is predominately a smoking-related cancer with a correspondingly high mutational load reflecting DNA damage from smoke carcinogens. Accordingly, this study characterized pre-neoplastic tissues as having the earliest molecular changes that activate immune sensing and response, whereas subsequent stages are distinguished by continual cell proliferation and accumulating somatic mutations that elicit an anti-tumor immune response. This in turn leads to high grade precancerous lesions with inherent immune suppression mechanisms just prior to progression to frank SCC. This study provides unique insights into early tumor-immune system interactions.

Collectively, these studies illustrate that not all genetic contributions to genome instability, to neoantigen generation, or to immune therapy responses are yet understood, and indeed that even when they are understood, these genetic contributions may not predict universal consequences for treatment outcomes.

## Implications for precision medicine

The implications of the studies described above on the use of immune checkpoint blockade therapies in the context of precision cancer medicine for patients with genomic instability are significant. First, they imply that a tissue-specific set of diagnostic assays may be important for determining which patients are most likely to respond to these drugs, which are expensive and which have significant associated toxicities for certain patients. These assays will need to be devised and tested on retrospective samples from clinical trials of each drug in each tissue site (assuming those trials and samples exist), in order to demonstrate their predictive potential, before they can advance to use in a randomized clinical trial that would confirm their role as a companion diagnostic. Second, this scenario significantly adds to the complexity and time-to-result for cancer patients, and invokes a higher cost of diagnostic testing that may not be reimbursed by insurance companies or governments with socialized medicine programs. Indeed, because most metastatic cancer patients have received multiple lines of therapy prior to checkpoint blockade therapy, many of which suppress the immune system to different degrees, and because they also have variable levels of disease burden that probably impact their response, we may never be able to predict immune checkpoint blockade response fully in every patient by using the same set of assays. Nonetheless, the standardization of NGS-based assays and analytical pipelines for determining TMB, neoantigen burden, and alterations to genes that impart genomic instability by studying both cancer and germline DNA is a worthwhile goal. In most cases, the same NGS data set can be used to evaluate the mutational status of important determinants of both immune status and neoantigen presentation, such as the mutational or gene-expression-based silencing of different *HLA* alleles or of beta-2-microglobulin (β_2_M), and of the activation of JAK/STAT pathways, which may also be indicative of existing or emergent resistance to checkpoint blockade therapy [87–91]. Specifying the optimal integration of diagnostic results from such NGS assays with those from conventional pathology-based assays (such as IHC-staining of CTLA-4, PD-1, and PD-L1 proteins) may drive a comprehensive evaluation of each patient that contributes to improved response prediction and may also indicate alternative therapeutic approaches when checkpoint blockade therapy is contraindicated.

## Conclusions and future directions

Genome instability in cancer results from a variety of genomic alterations, both germline and somatic. These alterations can be detected by different methods that reflect this variability in the underlying genes and their alterations, or can be simply evaluated by examining the downstream manifestation of the genomic defect using assays such as the detection of microsatellite instability. The recent studies reviewed here have begun to illustrate that not all types of genomic instability have the same impact when viewed in the context of immune cell recruitment or response to immune checkpoint blockade therapy. For example, even the widely accepted metric of TMB as a predictor of immune checkpoint blockade response is nuanced by other factors, both tumor intrinsic and extrinsic, that determine the likely response to immune modulatory drugs. Related to this conclusion, one pan-cancer study determined that frameshift alterations that result from insertion or deletion mutations produce strongly neoantigenic peptides and, overall, better predicted clinical responses to checkpoint blockade therapies [40].

Furthermore, there may be differences in mechanistic aspects of genomic instability that must be understood in the context of likely therapeutic response, such as the finding that cancers with hypermethylation-related MMRd appear to have reduced immune cell infiltration relative to mutation-related MMRd in several cancer types. These studies and others described in this review help to illustrate why TMB remains an imperfect predictor of therapeutic response to checkpoint blockade therapy as a standalone test across diverse tissue sites.

Evaluations of different methods to diagnose MSI-high cancers have demonstrated that NGS-based testing to detect microsatellite instability appears to be more sensitive than established methods such as PCR-based MSI assays. If designed correctly, NGS assays also can return information about resistance-associated alterations in immune response genes, overall TMB/neoantigen load, and different types of genomic alterations that may more accurately predict immunotherapy response. Driving the argument for the clinical benefit of such assays will require proper clinical trials that can ultimately provide a more confident prediction of response to expensive therapies and justify insurance reimbursement. Finally, several studies presented in this review emphasize that only the combination of correlative studies of banked tissues from clinical trials of different immune therapies, for which therapy response and outcomes are known, will enhance our understanding of the complex interplay of genomic instability, neoantigen generation, and immunomodulatory therapies. These studies, in turn, will inform the clinical management of cancer patients being treated with immunotherapy and will emphasize the gaps in our understanding of basic cancer immunity that require further elucidation.

## Abbreviations

DSB: Double-stranded break
HLA: Human leukocyte antigen
IHC: Immunohistochemistry
MCA: Methylcholanthrene
MHC: Major histocompatibility complex
MLH1hm: *MLH1* promoter hypermethylation
MMR: Mismatch repair
MMRd: Mismatch repair defect
MSI: Microsatellite instability
NGS: Next generation sequencing
SNV: Single nucleotide variant
TCGA: The Cancer Genome Atlas
TMB: Tumor mutational burden
